# Assessing Sarcopenia, Frailty, and Malnutrition in Community-Dwelling Dependant Older Adults—An Exploratory Home-Based Study of an Underserved Group in Research

**DOI:** 10.3390/ijerph192316133

**Published:** 2022-12-02

**Authors:** Lauren Swan, Niamh Martin, N Frances Horgan, Austin Warters, Maria O’Sullivan

**Affiliations:** 1 Department of Clinical Medicine, Trinity College, D02 PN40 Dublin, Ireland; 2 Older Person Services CHO9, Health Service Executive (HSE), D09 C8P5 Dublin, Ireland; 3 School of Physiotherapy, Royal College of Surgeons in Ireland (RCSI), University of Medicine and Health Sciences, D02 YN77 Dublin, Ireland

**Keywords:** older adult, home care, sarcopenia, frailty, malnutrition, physical activity

## Abstract

Background: Adults of advanced age, with functional dependency, socioeconomic disadvantage, or a need for home care, are expected to be at high risk of sarcopenia, frailty and malnutrition, yet are likely to be underrepresented in research. We aimed to explore the assessment of sarcopenia, frailty, and malnutrition in-home, and to describe the practicality of performing these assessments. Methods: Home-based health assessments and post-study feedback surveys were conducted among community-dwelling older adults ≥65 years in receipt of state-funded home care (*n =* 31). Assessments included probable sarcopenia [hand-grip strength (HGS), chair rise-test, and SARC-F case-finding tool], the Mini Nutritional Assessment (MNA), and the Clinical Frailty Scale (CFS). Results: The study group was of mean age 83.2 ± 8.2 years, 74% were female and 23% lived in socioeconomically disadvantaged areas. Almost all met the criteria for probable sarcopenia (94%, *n* = 29/31), were frail or vulnerable by the CFS (97%, *n* = 30/31), and over a quarter were at risk of malnutrition (26%, *n* = 8). Participants had low physical activity (71.0%, *n =* 22/31), with a mean daytime average of 11.4 ± 1.6 h spent sitting. It was possible to assess probable sarcopenia (by HGS and SARC-F, but not the chair rise test), malnutrition (MNA), and frailty (CFS). Home-based research was a complex environment, and unearthed significant unmet need, prompting referrals to health services (36%, *n* = 11), in addition to technology assistance. The majority of participants (93%) reported a willingness to partake in future research. Conclusions: Most community-dwelling older people in receipt of home support, assessed in this exploratory study, were at risk of probable sarcopenia, frailty, and low physical activity, with over a quarter were at risk of malnutrition. Our initial findings provide practical data for large scale studies and may inform the development of intervention studies aiming to support ageing in place.

## 1. Introduction

It is widely acknowledged that some population groups are not proportionally represented in public health and medical research [1]. Research in ageing observe lower participation among people with socioeconomic disadvantage, older age, and impaired physical function [1,2]. This is an important consideration when examining age-related conditions, such as sarcopenia and frailty, in older adult populations. To date, the evidence is predominately based on older adult cohorts under the age of 80 years, with the exception of the Newcastle 85+ study which has added to the evidence [3]. The present study focuses on older adults in receipt of government-funded home support, a population that includes a high proportion of adults aged 80 years and over, with moderate to high degree of frailty and a need for assistance with activities of daily living (ADLs) [4]. 

Sarcopenia, frailty, and malnutrition, independent predictors of greater healthcare utilisation, hospitalisation and mortality [5,6,7], are becoming increasingly prevalent in older adult populations [8]. Yet, these conditions are potentially reversible [9,10]. Sarcopenia, a condition characterised by the loss of muscle strength, mass, or function, contributes to functional limitations and mortality in older adults [5,7]. In 2018, the European Working Group on Sarcopenia in Older People 2 (EWGSOP2) updated their diagnostic criteria to include ‘probable sarcopenia’ defined by low muscle strength [11]. Probable sarcopenia is deemed present if low hand grip strength (women <16 kg and men <27 kg) or poor chair rise test performance (>15 s), markers of upper and lower extremity strength, is detected [11]. The introduction of probable sarcopenia is advantageous, as it can readily be measured in community-based settings, and once detected, is an appropriate timepoint to initiate interventions [11]. Frailty, a distinct entity to sarcopenia with overlapping properties, is a state of vulnerability with multi -system impairment and reduced capability to respond to external stressors [12]. While sarcopenia and frailty are linked to adverse health outcomes, both conditions are shown to be modifiable by targeted interventions, including physical activity and nutritional support [11,13]. 

Malnutrition, an important modifiable factor in sarcopenia and frailty [14], has a reported two-year incidence of 11% in older adult participants of The Irish Longitudinal Study of Ageing (*n =* 1841, mean age: 72 years) [15]. While the receipt of social support at home was found to significantly increase the risk of malnutrition in older adults [15]. Previous research with older adults in receipt of formal home support in Sweden (*n =* 353, mean age 82 years), reported that about half of this population were malnourished or at risk of malnutrition over 3-year follow-up [16]. The Mini Nutritional Assessment Short From (MNA-SF) represents a practical tool for the assessment of malnutrition risk and is recommended for use in community-based settings [17]. The MNA-SF requires body mass index, or a proxy, when determining malnutrition risk [17] and the practicality of collecting such measures in-home with dependant older adults remains unclear. 

Sarcopenia, frailty and malnutrition may co-exist in older populations [18]. A recent systematic-review and meta-analysis of sarcopenia, frailty and malnutrition reported a high overlapping prevalence in hospitalised older adult populations [18]. Previous findings, derived from administrative data, report a high prevalence of frailty (42%) and moderate functional dependency (48%) in older adults in receipt of formal home support services [4,19]. Similarly, we identified a 34% prevalence of probable sarcopenia in community-dwelling Irish older adults of mean age 66.9 years (*n =* 3342) [20]. Given frailty and sarcopenia predict future disability and mortality [5,12], potentially at-risk groups, such as those receiving routine care services, warrant formal screening and assessment [13]. In a recent study, the implementation of sarcopenia and frailty screening was assessed within an acute day-unit [21]. Dodds et al. suggest it is possible to implement routine sarcopenia assessment in an acute setting and identify a high prevalence of probable sarcopenia (84%) and frailty (66%) in a group of older adults with mean age 80.1 ± 7.7 years (*n =* 552) [21]. In community-based settings, sarcopenia, frailty and malnutrition are not routinely assessed or documented in this underserved older population group, and likely remain undetected and under-treated [22,23,24]. Little is known about older adults in the community, receiving formal home support. The introduction of probable sarcopenia in the updated EWGSOP2 guidelines may represent a simple practical approach to identifying older adults at high risk of sarcopenia in community-based settings and once detected, is deemed an appropriate timepoint to commence intervention [11]. We sought to assess probable sarcopenia, along with similarly practical measures of frailty and malnutrition among older adults in receipt of formal home care.

We conducted an exploratory home-based study among community dwelling older adults supported by formal home care, firstly, to assess markers of sarcopenia, frailty, malnutrition risk. We hypothesised that probable sarcopenia and frailty will be identified in a majority of the study group. Secondly, we aimed to examine the practicality of conducting in-home assessments for probable sarcopenia, frailty, and malnutrition risk with dependant older adults. 

## 2. Materials and Methods

### 2.1. Study Design and Population

**Figure 1:**
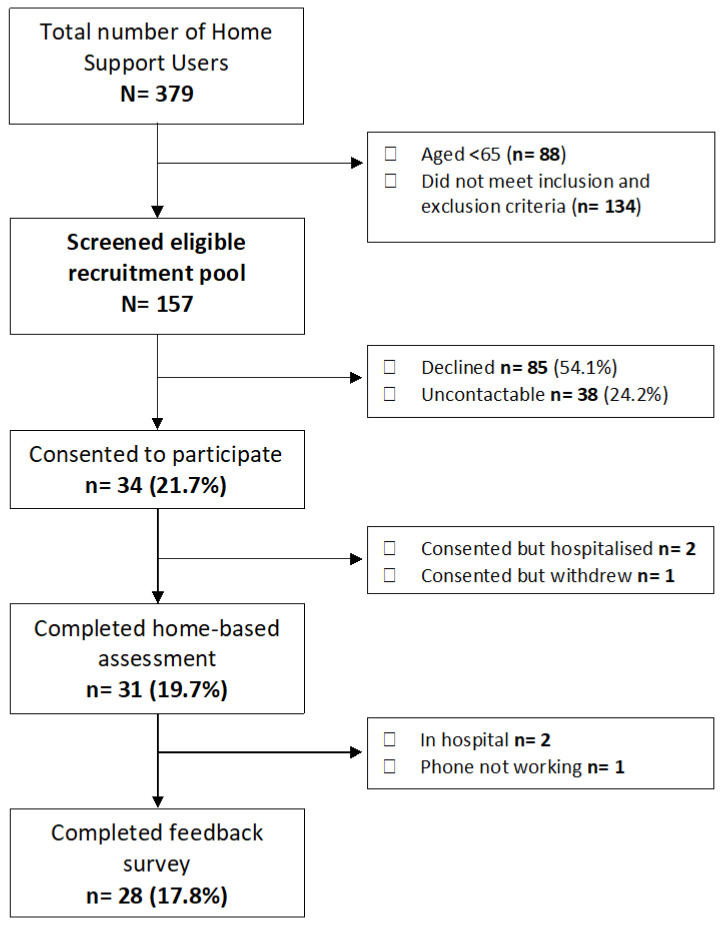
Flow diagram of participants in the home-based assessments and follow-up interviews.

This multi-method study included in-home assessments of sarcopenia, frailty and malnutrition and qualitative post-study feedback surveys with dependant older adults. Participants were invited to take part in the study based on the following inclusion criteria: aged 65 years and older, living at home and in receipt of state-funded home care services. Moderate to severe cognitive impairment or inability to provide informed consent and those in receipt of palliative care or medically unstable were excluded from the eligible recruitment pool (Figure 1). 

Convenience sampling was employed to recruit participants from a single government-funded not-for-profit home care organisation in Ireland acting as the study gatekeeper. To ensure diversity of participants, an organisation operating in areas of high and low socioeconomic deprivation was selected, with associations between younger age and receipt of formal home support in areas with high socioeconomic disadvantage previously reported [24]. Home support in Ireland is allocated based on a health professional assessment of need and is not income assessed. Study recruitment flyers were sent by post to service users meeting the inclusion and exclusion criteria by the study gatekeeper (*n =* 157). Participation was self-selecting, with the research team notified of an expression of interest by the study gatekeeper (*n =* 34). Written informed consent was obtained from all participants and ethical approval was granted by Trinity College Dublin’s Faculty of Health Sciences Ethics Committee (FREC/210909).

Assessments were completed in the participants home by a community nurse embedded in the health system and a health researcher (*n =* 31), between December 2021-March 2022. With permission, participants with communication difficulties were aided by family caregivers or through use of close-ended questions. Participant feedback surveys were conducted by telephone at the end of the study (March 2022), which included reasons for partaking in the study, future research, and preferred mode of contact (*n =* 28/31). All surveys were conducted by the study gatekeeper to maintain objectivity, recorded digitally and transcribed verbatim. Detailed fieldnotes were maintained by the research team members LS and NM to document completion rates of assessments and issues arising during each research visits, similar to Hall et al. [25]. All qualitative responses, including fieldnotes, were analysed using content analysis [26], to identify themes pertinent to the practicality of conducting in-home research with dependant older adults. An adverse event report form was maintained by the research team and the study gatekeeper.

### 2.2. Demographic Variables

Demographic information included age, gender, living alone and socioeconomic position (SEP). SEP was defined using three indicators: highest educational qualification, residential area socioeconomic deprivation, and Subjective Social Status (SSS). Educational attainment was classified as four groups: no formal qualifications, secondary school lower, secondary school upper and third-level qualification, as previously reported ^17^. The HP Pobal Deprivation Index [27], which uses data from the 2016 Irish Census, was used to determine the relative affluence or disadvantage of small residential areas (classified as affluent, marginally above average, marginally below average or disadvantaged). Subjective Social Status (SSS), was defined using the MacArthur scale of perceived position in the social hierarchy [28]. SSS scores ranged from 1–10 with lower scores indicating greater socioeconomic disadvantage. 

### 2.3. Assessing Sarcopenia, Frailty, and Malnutrition

We assessed probable sarcopenia according to the EWGSOP2 guidelines, using hand grip strength and chair rise test performance. Hand grip strength was measured using a Jamar Dynamometer (Chicago, IL, USA) and standardised using the procedure described by Roberts et al. [29]. Three measurements were taken from each hand with the maximum value used in analyses and gender-specific cut off values for low hand grip strength were applied: <16 kg for females and <27 kg for males [11]. Chair rise test performance was measured as the time taken to complete 5 chair rises using the protocol developed by Dodds et al. for home-based assessments with older adults [30]. Those unable to complete the chair rise test due to the use of a walking aid or without the use of arms were classified as having poor chair rise test performance [30]. As per EWGSOP2 guidelines, a time taken to complete 5 chair rises greater than 15 s was classified as poor chair rise test performance [11]. Additionally, the 5-item SARC-F tool assessing strength, assistance in walking, rising from a chair or bed, climbing stairs and falls was employed [31], with a cut-off score ≥4 indicating sarcopenia onset [11].

Frailty was assessed using the Clinical Frailty Scale (CFS) [32], a tool used to quantify the degree of disability from frailty through the assessment of independence in ADLs, physical function and cognition. Physical dependency was indicated by the Barthel Index, a 10-item scoring tool assessing assistance required to complete ADLs and classified by maximum dependency (score 0–4), high dependency (5–8), moderate dependency (9–11), mild dependency (12–19) and independence (20) [33]. Hospitalisation in the previous 12 months and attendance of a day centre was self-reported. 

The Mini Nutritional Assessment Short Form (MNA-SF), a validated 6-item tool used in the screening of malnutrition, produced a continuous score (0–14 points) and was classified as healthy nutritional status (12–14), at-risk of malnutrition (8–11) or malnourished (0–7) [34]. Anthropometric measurements, components of the MNA-SF, included weight (kg), height (m^2^) and calf circumference (cm), if the measurement of BMI was not feasible. Weight was measured using a portable digital scale, with height assessed using a portable stadiometer. BMI was classified by the WHO criteria: underweight (≥15–18.5 kg/m^2^), healthy weight (≥18.5–25 kg/m^2^), overweight (≥25–30 kg/m^2^) and obesity (≥30 kg/m^2^) [35]. We further applied the alternative ESPEN age-specific BMI cut offs for low BMI, recommended for use in the assessment of malnutrition risk: <20 kg/m^2^ if aged less than 70 years old and <22 kg/m^2^ if aged 70 years or older [36]. For the measurement of calf circumference, lower limb swelling (suspected oedema) was noted as present if identified visually and confirmed by the participant.

### 2.4. Health Variables

The International Physical Activity Questionnaire (IPAQ), was used to categorise activity in the previous 7 days as high, moderate, or low physical activity levels [37,38]. Behavioural factors including self-reported smoking and alcohol consumption. Self-reported health was rated as excellent, very good, good, fair or poor. Long-term conditions were assessed using the functional comorbidity index, producing a continuous count (range: 0–18) [39]. Number of medications prescribed per day was self-reported, confirmed with products or prescriptions within the home and excluded nutritional supplements and over the counter medications. Polypharmacy was defined by taking 5 or more medications per day and excessive polypharmacy was indicated by 10 or more prescribed medications [40]. 

### 2.5. Assessing the Practicality of Conducting In-Home Assessments

Completion rates for sarcopenia, frailty and malnutrition assessments were recorded. Pragmatic challenges to conducting in-home research were recorded in study fieldnotes. Post-study assessment, feedback surveys were conducted with participants to examine reasons for partaking in the study, future research, and preferred mode of contact (*n =* 28/31). Furthermore, the practicality of completing remote or online assessments, was examined using previously defined criteria for low technology readiness [41], (1) inability to use a telephone due to hearing impairment, (2) verbal communication difficulties (3) visual impairment causing difficulty in reading or watching television, (4) owning no internet-enabled devices and (5) no use of email, texting or internet in the previous month.

### 2.6. Data Analysis

Descriptive statistics are presented as proportions and mean ± standard deviation. The overlap between sarcopenia, frailty and malnutrition was visualised by means of Venn diagram. Fieldnotes and post-study feedback surveys were analysed by research team members AW and MOS. This included data pertinent to assessment completion rates, related challenges and older adults’ preferences for future in-home sarcopenia, frailty, and malnutrition research engagement. All analyses were performed using IBM SPSS Statistics V27 software. 

## 3. Results

### 3.1. Demographic and Health Characteristics of the Study Group

The study population (*n =* 31) was of mean age 83.2 ± 8.2 years, and the majority were female (74.2%), aged 80 years or older (67.7%) and lived alone (74%) (Table 1). Overall, 22.6% of participants lived in socioeconomic disadvantaged areas, with a similar proportion (25.8%) reporting no formal educational qualifications, indicative of disadvantaged SEP.

Low physical activity was reported among most participants (*n =* 22/31, 71.0%), with participants reporting a mean daytime average of 11.4 ± 1.6 h spent sitting. Polypharmacy (71.0%, *n =* 22/31), comorbidity (96.8%, *n =* 30/31) and reported hospitalisation in past 12 months (58.1%, *n =* 18/31) were prevalent. All participants received support with ADLs, and 87.1% had a Barthel Score indicative of mild physical dependency (*n =* 27/31). Of those with complete BMI measurement, based on WHO criteria, 71.4% had overweight or obesity (*n =* 20/28). No participant met the criteria for underweight BMI, based on WHO criteria. Low BMI, defined by ESPEN criteria, was identified in 10.7% of participants (*n =* 3/28). Based on MNA-SF calf circumference criteria for malnutrition risk, 4 participants (16.0%) had a calf circumference measurement less than 31 cm (Table 1). 

### 3.2. Identifying Probable Sarcopenia, Frailty and Malnutrition

**Figure 2:**
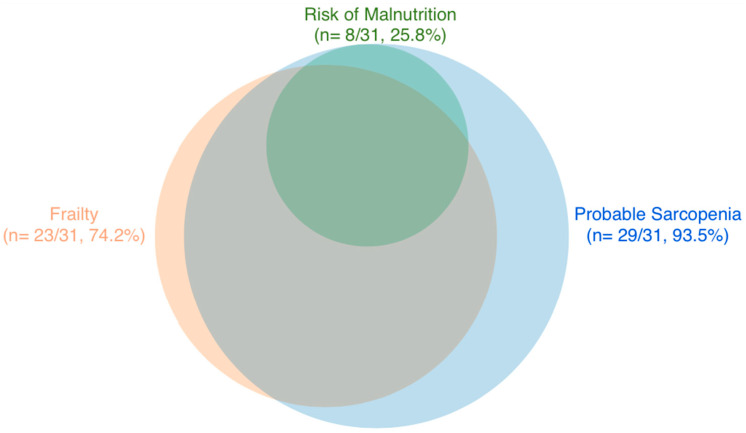
Venn diagram of overlap between probable sarcopenia, frailty and risk of malnutrition in dependant older adults (*n* = 31).

Probable sarcopenia was detected in almost all (93.5%, 29/31) participants as defined by EWGSOP2 criteria (Figure 2). Probable sarcopenia defined by low hand grip strength alone was identified in 61.3% (*n* = 19/31) of participants and in 90.3% by poor chair rise test performance (*n* = 28/31). The latter included 19 participants who were unable to complete the chair rise test due to an inability to stand without use of their arms (*n* = 3) or use of a walking aid (*n* = 16). 77.4% had a positive SARC-F score equal to a score of four or more (*n* = 24/31). 

Based on the Clinical Frailty Scale (CFS), most of the group were classified as either frail or vulnerable (*n* = 30/31, 97%). Specifically, 74.2% had frailty (*n* = 23/31), predominantly in the mild to moderate category (*n =* 21/31, 67.7%) (Table 1). According to the MNA-SF, over a quarter (25.8%, *n* = 8/31) were at-risk of malnutrition. There was a high degree of intersectionality in the screening of probable sarcopenia, frailty, and malnutrition risk, detected in 22.6% of the study population (*n* = 7/31) (Figure 2).

### 3.3. Completion Rates of In-Home Sarcopenia, Frailty, and Malnutrition Screening Tools

The SARC-F tool, CFS and MNA-SF had full completion rates (100%) in home. Probable sarcopenia assessed by hand grip strength and chair rise test were completed in 90% and 61%, respectively (Supplementary Table S1). In 3 cases only dominant hand readings were obtained due to neurological conditions or injury. It was not possible to conduct the chair rise test with individual who were unable to stand safely without the use of an aid or use of arms (*n* = 19). Where weight or height measurement was not viable, due to mobility limitations (*n* = 3, 9.7%), calf circumference measurements were available to calculate the MNA-SF, though measurement issues were noted for participants with suspected lower limb oedema (*n* = 6, 19.4%). The study group included participants (*n* = 7, 22.6%) with visual, hearing or communication impairments. This did not impact completion rates and with participants accommodated as appropriate, as outlined in the methods. 

### 3.4. Pratical Issues Relating to In-Home Research and Assessments

During the study visits, issues were raised by the participants relating to unmet needs. The fieldnotes showed that over a third of participants (*n* = 11/31, 35.5%) had one or more referrals made by the research team to health and social care services including public health nursing (*n* = 5), formal home support providers (*n* = 5), community physiotherapy (*n* = 2), occupational therapy (*n* = 1), disability services (*n* = 1), day centre (*n* = 1), as well as advising participants to speak with their GP (*n* = 4). In total, 15 referrals were made by the research team. Several issues related to complex social environments, including neighbourhood anti-social behaviour, social isolation, the absence of informal support, factors relating to complex family relationships including substance use. In addition, everyday issues raised, primarily related to technology use, were addressed, e.g., assistance with televisions, telephones, accessing mobile phone credit, email, and helplines for online services. The average duration of an in-home assessment visits was 78.5 min (range 40–150 min).

### 3.5. Potential for Future Engagement in Sarcopenia, Frailty and Manlutrition Research

Participants provided practical suggests for future in-home research with dependant community-dwelling older adults (Table 2). The study feedback survey showed that almost all the participants agreed to be contacted about future studies (*n* = 26, 92.9%) (Table 3). Participants stated a preference to be contacted by postal leaflets 46.4%, telephone 32.7% or via a primary care healthcare worker 17.9%. In exploring the possibility of using online assessments in future studies, we noted that indicators of technology unreadiness were common (74.2%, *n* = 23/31), for example not owning an internet-enabled device or no access to the internet (*n* = 17, 54.8%) (Table 1). Similarly, 61.3% reported no use of email, texting, or internet in the previous month (*n* = 19). 

## 4. Discussion

We assessed sarcopenia, frailty and malnutrition in a group of ADL-dependant community-dwelling older adults (mean age 83.2 ± 8.2) in receipt of formal home support. Most participants met the criteria for probable sarcopenia and frailty, and over a quarter were identified as at risk of malnutrition. Furthermore, low physical activity was prevalent, with participants reporting an estimated 11 daytime hours spent sitting. While these chronic conditions and patterns of sedentary behaviour have been reported in older people in acute settings and in longitudinal datasets [20,21,42,43], few studies have focused on older populations supported by formal home care [24]. The findings suggest opportunities for appropriate physical activity and dietary intervention to address sarcopenia, frailty, and malnutrition in this group.

Probable sarcopenia (94%, *n =* 29/31) and frailty (74%, *n =* 23/31) were present in a majority of the study population, although the sample size limits extrapolation of the findings. When assessed by low hand grip strength alone, probable sarcopenia was present in over half of participants (*n =* 61, 19/31) and almost all by poor chair rise test performance (90%, *n =* 28/31). In a relatively similar cohort to our study group, Dodds et al. [21] found similar patterns, with 84% of patients meeting the criteria for probable sarcopenia based on low hand grip strength, and 66% were frail based on Fried phenotype [21]. The latter was a large study of older adults attending an acute day unit in the UK (*n =* 552) with a mean age of 80.1 ± 7.7 years. In the present study, a majority of participants had a positive score (4+ points) on the SARC-F tool (77%, *n* = 24/31) suggesting high risk of sarcopenia onset and a prognostic indicator of mortality [31], mirroring patterns observed by Dodds et al. in an acute day setting (66%) [21]. The Newcastle 85+ study reported just under half of older adults aged 85 years and older have probable sarcopenia (48%), defined by low hand grip strength, in a population in which 22% report no dependency in ADLs [3]. Few studies have explored sarcopenia, frailty and malnutrition risk in populations of older age (mean age 80+ years) with dependency in ADLs. The findings build on previous work [21,43], suggesting older adults accessing aged care services, including formal home support, represent an important group for sarcopenia assessment and intervention strategies.

Previous research, examining frailty by the CFS in a population of older people in receipt of home support, classified 80% of the population as vulnerable or frail (*n =* 1312, mean age 82.1 ± 7.3 years) [19]. In contrast, a prevalence of frailty (24%) and pre-frailty (45%) was observed in younger participants of the Irish Longitudinal Study on Ageing (TILDA) (*n =* 3507, mean age 74 years) [6]. Importantly, the authors identified frailty as a significant predictor of higher health and social care service utilisation, specifically home care services [6]. The findings suggest older adults accessing formal care services are an important group for preventative intervention, with previous findings identifying case management and rehabilitative services as effective strategies in this group [44]. 

Similarly, use of formal home care services has been identified as a determinant of malnutrition risk in Irish older adults [15]. In the present study, over a quarter of participants were identified using the MNA-SF as at risk of malnutrition. The findings mirror those from a large multi-centre study of home care recipients in Germany (*n =* 878, mean age 78.5 ± 12.2 years), which found 20% of participants were at risk of malnutrition based on MNA-SF [16]. Lahmann et al. recommended the implementation of regular data collection and monitoring of weight as part of routine home care assessments [16], however the practicality of performing such in-home measurements with dependant older adults remained unclear. 

Designing research studies to include in-home assessments has been shown to reduce barriers to participation [45], however there is limited data on the use of pragmatic assessments and their completion rates in the home [46]. We found that assessment of probable sarcopenia (by hand grip strength), sarcopenia case-finding using the SARC-F tool, malnutrition (MNA) and frailty (CPF) was possible for all participants. The assessment of probable sarcopenia by chair rise test performance was possible for only 39% of participants, due to use of mobility aids or requiring the use of arms to stand. This is in line with previous research identifying 51% of home care clients in Finland were unable to complete this test in home-based assessments (*n =* 267, mean age 84.5 ± 5.2 years) [46]. Anthropometric components of the malnutrition assessment (MNA), specifically the measurement of BMI, presented challenges due to wheelchair use or reduced mobility. While the measurement of calf circumference was possible in these cases, suspected lower limb oedema, identified in 19% of the study population, may have inhibited the validity of this measure. Overall, it was possible to complete screening assessments for sarcopenia, frailty and malnutrition in home-with dependant older people, similar to findings in acute settings [21,43]. 

While an in-home study design with older people represented a complex research environment, it was not a barrier to completing the assessments, however, the research team unearthed considerable unmet need. Over a third of participants had referrals made by the research team to health and social care services, namely public health nursing, formal home support providers, community physiotherapy, occupational therapy services and disability services. Along with assisting with everyday issues including technology. In addition to high levels of physical dependency detected in this group, previous findings, mostly derived from administrative data, identify mental health conditions, social isolation and socioeconomic disadvantage [4,24]. Despite this complexity, the study population expressed a willingness to engage in research, with 93% agreeing to be contacted about future studies. In line with previous studies [47], altruistic factors were identified as motivators of research participation including a willingness to help others. Future research may be facilitated by embedding the assessments and research within routine primary care, or through the establishment of recruitment registries [48]. While technology may represent a useful tool, the findings show most of the study group were not technology ready. 

The assessments applied in the present study had high completion rates and described a high proportion of the study group as meeting the criteria for probable sarcopenia, frailty, malnutrition and physical inactivity. Although the sample size was low, limiting the generalisability of the findings, the initial observations suggest a future larger study is merited. For example, the identification of probable sarcopenia alone, is deemed sufficient evidence to initiate targeted interventions [11], specifically physical activity and nutritional support [11,13]. Ideally, future studies would assess frailty using other validated tools such as Fried phenotype [49]. Participants reported an average of 11 daytime hours spent sitting. Capturing this variable could be applied in future studies, or in routine practice, given that enabling older people to break-up the duration of sedentary time may be relevant in designing interventions appropriate to this group. While the introduction of mandatory screening strategies into existing older person services have been previously recommended [13,48], a tailored response including the delivery of timely treatment and interventions is required. Hendry et al., in a systematic review of interventions for frailty prevention, recommend their targeted delivery in high-risk frail community dwelling older adults [50]. Previous research examining the delivery of a physical activity program within formal home care services in Ireland reported improvements in physical function, however the high degree of frailty observed among this group required a customised approach [51]. Psychosocial factors, including social isolation and a lack of informal support, suggest a need for multi-dimensional approaches and support the potential of social prescribing initiatives for this population group [52].

This study has several strengths, including the application of validated questionnaires and screening tools with a study population of mean age 83 years, with frailty (74%) and socioeconomic disadvantage (26%), characteristics of underserved groups in research [1]. We collected data on sarcopenia, frailty and malnutrition, which could be practical additions to other routine health data collected in the delivery of formal home support services. Limitations include the small sample size, with the study designed to assess the practicality of conducting assessments in home-based research. Importantly, given the absence of e-health records and harmonised primary care data in Ireland, the present study represents the first to collect markers of sarcopenia, frailty and malnutrition in addition to demographic, socioeconomic and health data for dependant older adults accessing formal home support services in Ireland. 

## 5. Conclusions

Most community-dwelling older adults in receipt of home support, assessed in this exploratory study, met the criteria for probable sarcopenia, frailty and low physical activity, with over a quarter at risk of malnutrition. Conducting in-home assessments of probable sarcopenia, frailty and malnutrition risk was possible and had high completion rates. Although a group considered underserved by research, encouragingly, participants expressed a willingness to engage in future research. Our initial findings provide practical data for large scale studies and may inform the development of intervention studies aiming to support ageing in place.

## Figures and Tables

**Table 1:** Characteristics of the study population (*n =* 31).

	Overall (*n =* 31)
**Demographics**	
**Gender, *n* (%)**	
Female	23 (74.2)
Male	8 (25.8)
**Age, mean** **± SD (years)**	83.2 ± 8.2
**Age Categories, *n* (%)**	
65–69	1 (3.2)
70–79	9 (29.0)
80–89	16 (51.6)
90+	5 (16.1)
**Lives Alone, *n* (%)**	23 (74.2)
**Socioeconomic Position**	
**Educational Attainment, *n* (%)**	
Degree	4 (12.9)
Upper Secondary	13 (41.9)
Lower Secondary	6 (19.4)
No formal qualification	8 (25.8)
**Residential Socioeconomic Deprivation, *n* (%)**	
Affluent	12 (38.7)
Marginally Above Average	8 (25.8)
Marginally Below Average	4 (12.9)
Disadvantaged	7 (22.6)
**Subjective Social Status, mean** **± SD**	6.3 ± 1.8
**Screening Assessments**	
**Probable Sarcopenia, *n* (%)**	29 (93.5)
Low Hand Grip Strength, *n* (%)	19 (61.3)
Poor Chair Rise Test Performance, *n* (%)	28 (90.3)
SARC-F Positive Score, *n* (%)	24 (77.4)
**Clinical Frailty Scale (CFS), *n* (%)**	
CFS3—Pre-frail	1 (3.2)
CFS4—Vulnerable	7 (22.6)
CFS5—Mild Frailty	11 (35.5)
CFS6—Moderate Frailty	10 (32.3)
CFS7—Severe Frailty	2 (6.5)
**Mini Nutritional Assessment Category, *n* (%)**	
Normal nutritional status	23 (74.2)
At risk of malnutrition	8 (25.8)
**Body Mass Index (BMI, kg/m^2^) ^a^**	
**World Health Organization (WHO) critieria, *n* (%)**	
Underweight (≤18.5 kg/m^2^)	0 (0.0)
Healthy weight (≥18.5–25 kg/m^2^)	8 (28.6)
Overweight (≥25–30 kg/m^2^)	13 (46.4)
Obesity (≥30 kg/m^2^)	7 (25.0)
**ESPEN criteria, *n* (%)**	
**Low BMI ^b^**	3 (10.7)
**Calf Circumference, mean** **± SD (cm) ^c^**	34.6 ± 4.4
Suspected oedema in lower limbs, *n* (%)	6 (19.4)
**MNA Calf Circumference Category, *n* (%) ^c^**	
Calf circumference < 31 cm	4 (16.0)
Calf circumference ≥ 31 cm	21 (84.0)
**Lifestyle Factors**	
**Physical Activity Level, *n* (%)**	
Low	22 (71.0)
Moderate	8 (25.8)
High	1 (3.2)
**Daytime hours spent sitting daily, mean** **± SD**	11.4 ± 1.6
**Smoking status, *n* (%)**	
Never smoked	14 (45.2)
Past smoker	16 (51.6)
Current smoker	1 (3.2)
**Alcohol consumer, *n* (%)**	12 (38.7)
**Health Factors**	
**Self-rated Health, *n* (%)**	
Excellent	0 (0.0)
Very Good	4 (12.9)
Good	15 (48.4)
Fair	11 (35.5)
Poor	1 (3.2)
**Long-term conditions, *n* (%)**	
0	1 (3.2)
1	0 (0.0)
≥2	30 (96.8)
**Mental health conditions, *n* (%)**	10 (32.3)
**Polypharmacy, *n* (%)**	
Prescribed ≥ 5 medications	22 (71.0)
Prescribed ≥ 10 medications	7 (22.6)
Mean number of medications ± SD	7.0 ± 2.9
**Barthel Index Score, mean** **± SD**	15.0 ± 3.5
**Barthel Index Category, *n* (%)**	
Maximum dependency	1 (3.2)
High dependency	1 (3.2)
Moderate dependency	2 (6.5)
Mild dependency	27 (87.1)
Independent	0 (0.0)
**Health and Social Care Utilisation**	
In receipt of state-funded home support services	31 (100.0)
Hospitalisation in previous 12 months, *n* (%)	18 (58.1)
Attends day centre, *n* (%)	3 (9.7)
**Technology**	
**Technology Unreadiness, *n* (%)**	
Hearing difficulties	4 (12.9)
Visual impairment	2 (6.5)
Communication difficulties	1 (3.2)
No internet/internet-enabled devices	17 (54.8)
No use of email, texting, or internet in previous month	19 (61.3)

^a^ Missing data *n* = 3 (9.7%). ^b^ Low BMI was defined as <20 kg/m^2^ in those aged less than 70 years old and <22 kg/m^2^ if aged 70 years or older. ^c^ Excludes those with suspected lower limb oedema (*n =* 6, 19.4%). Abbreviations: SD, Standard Deviation; cm, centimetres; MNA, Mini Nutritional Assessment.

**Table 2:** Learnings for future in-home research engaging community-dwelling older adults with dependency based on participant feedback (*n* = 28).

Learning Points	Supporting Data	Considerations for Future Research
**Engaging Older Adults in Research**		
Recruitment: Involving community healthcare staff, eg home care workers and Public Health Nurses (PHNs), with existing relationships in the population of interest may be an effective approach to enaging older adults in research	*“If you wanted more people, you should be asking the [home care workers] to feedback to the people they’re visiting about the research study”* P002	Community representatives may enhance engagement of older adults in research
Participants were concerned about their physical capabilities prior to the research visit	*“I was worried that there would be lots that I couldn’t do. I use a three-wheeler to stand and a stick to get around so I was worried that I wouldn’t be physically able for it”* P021	Prospective participants may be concerned about their physical capability to engage in research. Researchers may need to alleviate concerns through highlighting the adaptablility of assessments and their voluntary nature. There is a need to develop plans for resasonable accomodations, including adaptable and accessible assessments.
Older people, including those supported to live independently through home care, are willing to participate in research	Almost all participants (93%) agreed to be contacted about future studies	Future studies should actively aim to engage older adults currently underserved in health research
Participants recommended promoting the benefits of research	*“I think you need to get across the long-term benefit of research to old people. You need to tell them that this is what we have to do if we want to improve care for people in the community”* 003	There is a need for greater awareness around the value, potential benefits and applications of research to improve engagement in underserved groups.
**Conducting in-home Research**		
Conducting research in the participants’ homes is complex, and may identify considerable unmet need.	36% of participants (*n =* 11) had one or more referrals made on their behalf to health and social care services following the research visit. The opportunity for a nurse visit was reported as motivating factor to engage in the research study by 25% of participants.	Researcher teams should be aware of their duty of care, including the importance of having strategies in place to respond to identified unmet needs or other potential issues arising (eg safeguarding) during research visits. The inclusion of an experienced healthcare professional, embedded within the primay care system, within the research team was essential in our study to action identified unmet needs.
There was additional time associated with the completion of in-home research visits. In some cases, this was due to assistance with a range of everyday tasks inside the home such as providing support with technology.	The average duration of an in-home assessment visit was 78.5 min (range 40–150 min).	Researchers should acknowledge the additional complexity associated with conducting studies in a participant’s home, and allocate sufficient time, flexibility and resources. It’s their homes, into which we, as reseachers, have been invited.
There was a need to adapt our approach to completing assessments for participants with communication difficulties, such as including an informal caregiver in the research visit, with the participant’s permission.	The study group included participants (*n =* 7, 22.6%) with visual, hearing or communication impairments.	Conducting research with older adults who may additional communication needs requires an adaptable and person-centred approach

**Table 3:** Participant Feedback on Engaging in Future Research Studies (*n =* 28).

	*n* (%)
Motivation for partaking in this study	
Willingness to help others	11 (39.3)
Concerned or interested in the topic, i.e., muscle strength	8 (28.6)
Opportunity for a nurse visit	7 (25.0)
Family encouragement	1 (3.6)
Other reason	1 (3.6)
Agreed to be contacted about future studies	26 (92.9)
Preferred contact method for research	
Postal leaflet	13 (46.4)
Telephone	9 (32.1)
Community healthcare worker	5 (17.9)
Email	1 (3.6)

Missing Data (*n*, %); (*n =* 3, 9.7%).

## Data Availability

Data are available upon request from the corresponding author L.S. at swanla@tcd.ie.

## Supplementary Materials

The following supporting information can be downloaded at: https://www.mdpi.com/article/10.3390/ijerph192316133/s1, Supplementary Table S1: Completion Rates and reason for non-completion of home-based assessments of probable sarcopenia, frailty, and malnutrition (*n* = 31).
