# Supplementary material for: Assessing Sarcopenia, Frailty, and Malnutrition in Community-Dwelling Dependant Older Adults—An Exploratory Home-Based Study of an Underserved Group in Research
Source: Int J Environ Res Public Health. 2022 Dec 2;19(23):16133. doi: 10.3390/ijerph192316133 (PMC9736424; doi:10.3390/ijerph192316133)
Supplement: Supplementary file 1 [file ijerph-19-16133-s001.zip › ijerph-1960521-supplementary.pdf]

Supplementary Table S1: Completion rates and reason for non-completion of home-based assessments of probable sarcopenia, frailty and malnutrition (n = 31)

Assessment	Possible to complete n, (%)	Reasons for incompletion
**Probable Sarcopenia Screening**		
SARC-F	31 (100.0)	
Hand grip strength test [a]	28 (90.3)	• Neurological condition (n= 2)<br>• injury/pain (n= 1)
Chair rise test	12 (38.7)	Could not stand safely without the use of:<br>• an aid (n= 16)<br>• arms (n= 3)
**Frailty Screening**		
Clinical Frailty Scale	31 (100.0)	
**Malnutrition Risk**		
MNA	31 (100.0)	
BMI (height/weight)	28 (90.3)	• wheelchair user (n= 2)<br>• reduced mobility (n= 1)
Calf circumference [b]	25 (80.6)	• lower limb oedema (n= 6)

[a] Defined by 3 measurements obtained for both dominant and non-dominant hands

[b] Used to compute MNA-SF score in the absence of valid BMI data. Excludes participants with lower limb oedema (n= 6, 19.4%)
